# New Biomarkers and Diagnostic Tools for the Management of Fever in Low- and Middle-Income Countries: An Overview of the Challenges

**DOI:** 10.3390/diagnostics7030044

**Published:** 2017-07-21

**Authors:** Camille Escadafal, Christian Nsanzabana, Julie Archer, Violet Chihota, William Rodriguez, Sabine Dittrich

**Affiliations:** FIND (Foundation for Innovative New Diagnostics), 1202 Geneva, Switzerland; camille.escadafal@finddx.org (C.E.); christian.nsanzabana@finddx.org (C.N.); julie.archer@finddx.org (J.A.); violet.chihota@finddx.org (V.C.); brod19@gmail.com (W.R.)

**Keywords:** biomarker, fever, febrile illness, resource-limited setting, diagnostic, low- and middle-income countries, challenges, diagnostic tests

## Abstract

A lack of simple, inexpensive, and rapid diagnostic tests for febrile illnesses other than malaria leads to overtreatment with antibiotics for those who test negative for malaria, and contributes to the global rise in antimicrobial resistance. New tests for the detection of host biomarkers provide promising tools to differentiate bacterial from non-bacterial infections in febrile patients. However, most available biomarker tests are not currently used in resource-limited settings, and very few evaluations have been performed in low- and middle-income country populations with non-severe febrile illness. As a result, our knowledge of the performance of these tests in settings with high prevalence of infectious and poverty-related diseases such as malaria, HIV, malnutrition and intestinal parasites is poor. This paper describes challenges faced during the process of getting to an approved test, including difficulties in selecting the most appropriate fever biomarkers; suitable study designs and sites for test evaluations; lack of available reference tests to evaluate the performance of new tests; and lack of clear regulatory pathways to introduce such tests. As many new biomarker assays are in development, understanding these challenges will better enable those working in this area to address them during product development.

## 1. Background 

Acute febrile illnesses are a major cause of global mortality and morbidity. Although reliable data are scarce, estimates suggest that more than 180 million fever episodes occur annually in young children in sub-Saharan Africa alone, with most cases remaining undiagnosed and, often, inappropriately treated [1]. Unfortunately, similarly well-established data are not available for adults with fever. While fevers can result from both infectious and non-infectious causes [2], available data support the assumption that the predominant causes of fever in the tropics are infectious. 

Several recent initiatives to identify the most common infectious causes of non-malarial fevers in Africa [3,4], Asia [5,6] and Latin America [7] have demonstrated wide variation between regions, making empiric management and antibiotic selection difficult. However, with the notable exception of the malaria rapid diagnostic test (RDT), there are no simple, cheap, pathogen-specific tests to provide actionable results to health workers for the appropriate management of common febrile illnesses [8]. Among paediatric febrile patients who presented to health facilities in sub-Saharan Africa, an estimated 57% remained undiagnosed following a negative result from a malaria RDT [1]. An increasing body of evidence shows that the widespread use of malaria RDTs, while substantially decreasing the inappropriate use of anti-malarial medicines, has inadvertently contributed to the growth in antimicrobial resistance through the over-prescription of antibiotics in malaria-negative patients [9]. This development is especially worrying as malaria cases decrease globally, particularly in Southeast Asia, where antimicrobials are easily accessible over the counter. According to the 2014 World Malaria Report [10], more than 142 million malaria case investigations were negative for malaria, but without other tests to guide targeted treatment; many of these cases will be treated empirically. Based on available information, it is safe to assume that some proportion of these cases will have left health facilities with unnecessary antibiotics, and others may have gone on to purchase over-the-counter antibiotics elsewhere in the absence of other treatment. There is therefore an urgent need for improved diagnostics to guide the treatment of febrile, malaria-negative patients. 

One promising approach has been the use of host biomarkers to triage patients without severe illness to distinguish bacterial from non-bacterial infections, and guide the determination of who would most benefit from antibiotics. At a minimum, a biomarker-based triage test to distinguish between bacterial and non-bacterial infections could help to drive down the overuse of broad-spectrum antibiotics for non-bacterial infections and guide case management. An infection severity test could further assist with appropriate clinical decision-making. Such tests would support health care workers in clinical management, enable better care, and reduce patient exposure to unnecessary antibiotics and their side effects. The global reduction of unnecessary antibiotics is one important cornerstone of the fight against the rise of antimicrobial resistance [11]. 

However, there has been little evidence generated to support the use of biomarkers for fever triage in low- and middle-income countries (LMICs) [12,13]. Few studies of appropriate biomarkers have been conducted, especially in tropical regions, and there is a lack of evidence of host biomarker usefulness where low nutritional status and co-infection with parasites or other pathogens could undermine their effectiveness [8,12]. Several new multiplexed biomarker assays based on protein or transcriptomic biomarkers [14,15,16,17,18] have been developed in the last few years; none have been validated or made commercially available in LMICs. Alongside these efforts to identify new combinations of biomarkers, research groups in Southeast Asia have been exploring the use of C-reactive protein (CRP) to differentiate bacterial infections from non-bacterial infections in Southeast Asia, albeit with limited specificity [19,20,21]. 

To support and accelerate the next phases of the development and implementation of biomarker triage tests, this paper identifies several challenges and knowledge gaps, and offers some proposed solutions. We hope this overview of important considerations will help researchers, developers and their partners to design conclusive clinical trials to inform global and national health policy, and spur the development of new diagnostic tools designed for resource-limited settings around the world. It will not be possible to implement these tests in LMICs unless the challenges are well understood and considered prior to product development and demonstration trials.

## 2. Availability of Fever Triage Tests 

Despite the burden of acute febrile illnesses in LMICs, there is little evidence of the use of biomarkers for fever triage in those countries [12]. A recent review found that a total of 112 unique host biomarkers were reported in 59 studies since 2011, but only 19% of the studies included patients from LMICs [8]. The markers showed varying levels of specificity and sensitivity in the differentiation of bacterial from non-bacterial infections; a combination of different markers tended to provide slightly higher sensitivity and specificity compared to the use of a single marker alone [8]. While commercial assays combining some of these biomarkers are available, none are specifically designed to differentiate bacterial from non-bacterial infections, and few are suitable for wide use in low-resource settings. 

The need for a simple and rapid triage test to differentiate bacterial from non-bacterial infections to guide clinical case management has been recognized by global health stakeholders, including those concerned about the inappropriate use of antibiotics and its contribution to the alarming growth in antimicrobial resistance. In 2016, a working group convened by the World Health Organization (WHO), the Médecins Sans Frontières (MSF) Access Campaign, ReAct, and FIND published the target product profile of such a triage test to guide diagnostics researchers and developers [12]. 

While no available assays meet the profile of preferred product characteristics for LMICs, there are several exciting new products emerging from the development pipeline. Two new assays developed for differentiating bacterial from non-bacterial infections have recently received regulatory clearance in Europe. The ELISA-based ImmunoExpert™ assay (MeMed Diagnostics, Tirat Carmel, Israel) measures three different host proteins: CRP, IP-10 and TNF-related apoptosis-inducing ligand (TRAIL) [17,22]. This combination includes one inflammatory marker predictive for bacterial infections (CRP) and two proteins that have been shown to be upregulated in viral infections (TRAIL and IP-10). Clinical studies in selected settings in Israel demonstrated that the combination assay is more sensitive and specific than using each protein separately [16]. The ELISA format has received the CE mark (indicating compliance with the European Union (EU) Directive 98/79/EC, a widely used pre-market milestone for diagnostics developers). This assay is now commercially available in Europe, and the same company is developing a simple rapid test format. A second, simple CE-marked point-of-care assay to differentiate viral from bacterial acute febrile respiratory infection, FebriDx^TM^ (RPS Diagnostics, Sarasota, FL, USA), is a semi-quantitative test that combines the assessment of CRP and the myxovirus resistance protein 1 (MxA), a marker for viral infection, and provides results within 15 min [23]. Two other biomarker-based assays also recently received regulatory approval for use in febrile illness (SeptiCyte^®^, Immunexpress, Seattle, WA, USA; and the PSP IVD capsule for the abioSCOPE^®^, Abionic, Epalinges, Switzerland). These assays show promising early results, but they target sepsis and indicate illness severity rather than differentiating bacterial from non-bacterial infections. As with ImmunoExpert™ and FebriDx^TM^, these assays have not yet been evaluated in LMICs [8]. 

In the absence of extensive laboratory testing capacity to enable a diagnosis in most LMICs, integrated guidelines for management of childhood illness (IMCI) were established in 1997 to provide an evidence-based approach to case management, and decrease child mortality and morbidity [24]. These guidelines have since been updated to include malaria RDTs and promote the test-and-treat policy to guide the use of anti-malarials. In a recent review from WHO, it was estimated that to date, 90 of 97 LMICs have incorporated the IMCI guidelines into their national child health strategies [25]. However, the malaria RDT remains the only feasible diagnostic test integrated into the general algorithm of the IMCI guidelines. 

## 3. Challenges 

There are several important challenges on the path between late stage development and validation to implementation. These potential obstacles are important considerations for developers and global health decision-makers. 

### 3.1. Lack of Reference Tests for Comparative Analysis

In order to assess a new biomarker assay for its ability to differentiate bacterial from non-bacterial infections, it is critical to establish a definitive microbiologic diagnosis in all study subjects and define reference standard methods. However, there is no single reference test available as a comparator to assess diagnostic accuracies. The cause of fever must be identified with as much certainty as possible using a range of diagnostic tests. Moreover, even after having completed an extensive battery of microbiological tests, a large proportion of fevers will remain undiagnosed [3,4,5,6]. The findings from non-malarial fever studies across continents underline the limits to relying solely on microbiological testing to categorize bacterial and non-bacterial infections. Kapasi et al. (2016) [8] and subsequent studies highlight the great methodological variation between more than 60 fever studies, and the different approaches taken to deal with the lack of a single gold standard reference test. In a few studies, the bacterial versus non-bacterial classification was based only on the clinical assessment of the attending physician (3.2%, 2/63), and in others, patients were classified based on microbiological data alone (19%, 12/63). Many studies applied a combination of clinical assessment and microbiology laboratory data, as well as other laboratory markers, such as white blood cell counts (25.4%, 16/63). In 27% (17/63) of the studies, all clinical and laboratory data were reviewed by a panel of clinicians to decide on a consensus final diagnosis. The latter approach has also been used by a number of recent studies in patients from Europe, Israel and the U.S. that evaluated new multiplexed biomarker assays [16,17]. The issue is further compounded by the challenges associated with choosing not only the right pathogen to test for (guided by epidemiology, seasonality and study population) but also the right diagnostic approach (direct versus indirect testing, guided by time of presentation, sample type and clinical syndrome). 

While molecular detection methods such as polymerase chain reaction (PCR) have high analytical sensitivity and specificity, they are not always suited to determining the cause of illness and may have a reduced diagnostic sensitivity. For example, when comparing the PCR results from the respiratory or stool samples of febrile patients with those from a control group with no symptoms, it is not always easy to pinpoint the cause of infection, as similar organisms can be identified in the disease and healthy control groups [26,27,28]. This further supports the need to include clinical symptoms and, where appropriate, quantitative assessments to differentiate between colonization, carriage and infection causing disease. A similar challenge exists with the use and interpretation of single titre serology results, which in many settings might be indicative of past or recent infection but do not confirm an acute infection (for example, *Salmonella typhi*, *O. tsutsugamushi*).

To overcome these intrinsic technical challenges and to find a comparable approach between different studies, the combination of clinical and microbiological data reviewed by an independent panel could provide a workable compromise between quality and quantity of diagnostic data when studies are performed in different LMICs settings. 

### 3.2. Lack of Regulatory Clarity

Another critical issue is the lack of clear guidance from regulatory bodies for tests that differentiate bacterial from non-bacterial infections based on host biomarkers. There is currently no clear regulatory pathway for such tests to be approved for commercial use by the WHO Prequalification (PQ) of in vitro diagnostics programme [29], which promotes and facilitates access to safe, appropriate, affordable and good-quality in vitro diagnostics. Global health procurement agencies generally require diagnostic devices destined for LMICs to have been prequalified by WHO or another stringent regulatory body. However, the WHO PQ programme does not yet have a specific pathway for approving tests intended to differentiate bacterial from non-bacterial infections, and it may take time to develop a programme to approve such tests. 

In the absence of global guidance, one alternative would be to submit via the U.S. Food and Drug Administration (FDA) regulatory pathway. Prior to 2016, FDA guidance on the intended use of an in vitro diagnostic based on host markers to differentiate bacterial from non-bacterial infections was limited. However, in 2017 the FDA cleared the expanded use of a test for a single host marker, procalcitonin (PCT), in the initial triage decision for patients presenting with lower respiratory tract infection (LRTI). FDA clearance for PCT’s expanded use was supported largely by a meta-analysis of the available literature, a series of pragmatic trials of PCT, which collectively show efficacy (i.e., reduced antibiotic use based on PCT levels) without a compromise of safety (i.e., more deaths in the non-antibiotic group) [30]. This decision by the FDA suggests that routine submission based on the results of pragmatic trials of host biomarkers, comparing reduction in antibiotic use against safety, is a viable pathway for host biomarker-based triage tests—so long as the studies focus on a well-defined clinical condition, like LRTI.

### 3.3. Co-Morbidities in Low- and Middle-Income Countries

The biomarkers CRP and PCT have been in use for many years [8], and while they have been widely evaluated in European and U.S. populations, when first evaluated in Africa or Asia it became apparent that the expression levels of the molecules were influenced by co-morbidities such as HIV, malaria, parasites and malnutrition [20,31,32,33]. These confounding parameters can skew levels of the assessed biomarker, which could lead to inappropriate therapy. For example, levels of biomarkers such as CRP may be elevated in malaria cases, which could lead to overtreatment with antibiotics and under-treatment with anti-malarials. By contrast, malnutrition can cause relevant biomarkers to be particularly low, leading to the underdiagnosis and inappropriate management of potentially serious bacterial infections. These findings highlight the need to evaluate biomarkers in target populations in intended settings of use, and for the establishment of regionally-specific biomarker ranges and guidance for test interpretation. This should be taken into consideration at the diagnostic product development, national registration, implementation planning and policy development stages. 

### 3.4. Lack of Compatibility of Clinical Trial Needs with Intended Use Cases

When defining the study population of a biomarker evaluation study, it is crucial to keep the final use case of the diagnostic tool in mind. During the development of target product profiles for diagnostics for febrile illness in LMICs, the expert working group convened by WHO, MSF, ReAct and FIND defined an acceptable target population as “Children with non-severe, non-malarial acute fever presenting at health facilities” and the most desirable as “Total febrile population (including neonates) presenting with fever” [12]. The working group prioritized these populations at the most peripheral levels of the health system, due to the need for a simple triage test for non-severe patients at the point of first contact with a health worker, with the aim of reducing the unnecessary use of antibiotics at peripheral levels of the health system, where diagnostic capacities are negligible and empiric treatment is most common [9]. To evaluate a diagnostic test in this target population, academic, non-governmental and clinical research organizations are faced with the challenge of finding a compromise between available capacity (most often found in better-resourced cities) and access to the desired target populations (in the lowest resourced, often rural, areas). Structures such as regulatory authorities and institutional review boards that have key roles in the approval of trials may be non-existent or non-functional, or may lack the skills to critically review such protocols. The challenge is further augmented by the need for good laboratory and data capturing facilities to perform basic reference testing and sample processing/storage, and collect a minimal clinical dataset to allow the classification of enrolled subjects (non-bacterial/bacterial) to enable data analysis. Particularly given the confounders in different sub-populations (e.g., malnutrition, co-infections, as noted above), trial design is important if the study is to yield data on the utility of a test in key populations and different regions. Given the need for evaluation studies, particularly at a time when promising new biomarker assays are emerging [14,15,16,17,18,23,34], it will be important to develop guidance documents to help researchers, developers, regulatory authorities, institutional review boards and nongovernmental organizations in the planning of biomarker trials. Further, in line with what Sweeney and Khatri (2017) [35] propose in their review, samples, as well as knowledge/data-sets, obtained in clinical evaluations in relevant areas should be made freely available in open-access, centralised repositories, as has been done for gene expression studies in sepsis or to support the product development of TB diagnostic products [35,36]. This approach will enrich the overall data landscape and leverage resources used for clinical trials and researchers all across the world. 

## 4. Summary

There is an urgent need for simple, inexpensive, biomarker-based triage tests to distinguish between bacterial and non-bacterial infections, and for simple severity tests to support the triage process and case management of febrile patients in LMICs. Rapid and simplified pathogen identification tests for severe illness are also a priority. Evaluation of the performance of new and existing biomarker assays in sub-Saharan Africa, Asia and Latin America will be an important first step to fast-track the identification and approval of appropriate triage tests for each region. The development of new, region-specific multiplexed tests that combine pathogen identification with a biomarker-based identification of bacterial infections would be the logical next step. Such tests would provide actionable results for health care workers in low-resource settings and reduce the overuse of antibiotics. FIND is taking one step in this direction with its fever diagnostics portfolio, which includes multiplex assays for pathogens, pathogens plus biomarkers, and biomarkers alone. 

With many recent initiatives and incentive prizes (the UK’s Longitude Prize is the most prominent example), it is likely that innovative new solutions to differentiate bacterial from non-bacterial infections are on the horizon. In order to speed up the time to implementation, now is the time to identify challenges and work collectively on strategies to ensure the generation of strong validation data that can be used by regulatory authorities and normative agencies. Subsequently, approved biomarker tests will need to be integrated into clinical algorithms, such as the Integrated Management of Neonatal and Childhood Illnesses (IMNCI) [24,37] or its electronic derivatives [38,39,40]. Preliminary research data suggest that the combination of biomarker tests with e-algorithms could reduce the overuse of antibiotics by nearly 40% [41], which, if maintained in real-world use, would have a significant impact on the global crisis of antimicrobial resistance. 

While addressing the technical challenges faced in biomarker diagnostics development, it is important not to lose track of other factors required to ensure sustained use of these tests, including trained laboratory technicians and laboratory infrastructure. Parallel work must also be done in the areas of education and behaviour change regarding antibiotic prescription and use, clinical and laboratory capacity, policy change and sustainable payment structures that ensure uptake in health systems with limited budgets. 
